# Large-scale data mining pipeline for identifying novel soybean genes involved in resistance against the soybean cyst nematode

**DOI:** 10.3389/fbinf.2023.1199675

**Published:** 2023-06-20

**Authors:** Nour Nissan, Julia Hooker, Eric Arezza, Kevin Dick, Ashkan Golshani, Benjamin Mimee, Elroy Cober, James Green, Bahram Samanfar

**Affiliations:** ^1^ Agriculture and Agri-Food Canada, Ottawa Research and Development Centre, Ottawa, ON, Canada; ^2^ Department of Biology and Ottawa Institute of Systems Biology, Carleton University, Ottawa, ON, Canada; ^3^ Department of Systems and Computer Engineering, Carleton University, Ottawa, ON, Canada; ^4^ Agriculture and Agri-Food Canada, Saint-Jean-sur-Richelieu Research and Development Centre, Saint-Jeansur-Richelieu, QC, Canada

**Keywords:** soybean cyst nematode, computational biology, protein–protein interactions, bioinformatics, SCN resistance

## Abstract

The soybean cyst nematode (SCN) [*Heterodera glycines* Ichinohe] is a devastating pathogen of soybean [*Glycine max* (L.) Merr.] that is rapidly becoming a global economic issue. Two loci conferring SCN resistance have been identified in soybean, Rhg1 and Rhg4; however, they offer declining protection. Therefore, it is imperative that we identify additional mechanisms for SCN resistance. In this paper, we develop a bioinformatics pipeline to identify protein–protein interactions related to SCN resistance by data mining massive-scale datasets. The pipeline combines two leading sequence-based protein–protein interaction predictors, the Protein–protein Interaction Prediction Engine (PIPE), PIPE4, and Scoring PRotein INTeractions (SPRINT) to predict high-confidence interactomes. First, we predicted the top soy interacting protein partners of the Rhg1 and Rhg4 proteins. Both PIPE4 and SPRINT overlap in their predictions with 58 soybean interacting partners, 19 of which had GO terms related to defense. Beginning with the top predicted interactors of Rhg1 and Rhg4, we implement a “guilt by association” *in silico* proteome-wide approach to identify novel soybean genes that may be involved in SCN resistance. This pipeline identified 1,082 candidate genes whose local interactomes overlap significantly with the Rhg1 and Rhg4 interactomes. Using GO enrichment tools, we highlighted many important genes including five genes with GO terms related to response to the nematode (GO:0009624), namely, *Glyma.18G029000*, *Glyma.11G228300*, *Glyma.08G120500*, *Glyma.17G152300*, and *Glyma.08G265700*. This study is the first of its kind to predict interacting partners of known resistance proteins Rhg1 and Rhg4, forming an analysis pipeline that enables researchers to focus their search on high-confidence targets to identify novel SCN resistance genes in soybean.

## 1 Introduction

Cultivated soybean (*Glycine max* (L.) Merr.) is a valuable crop worldwide, regularly used in food, feed, and fuel. Soybean is also an important partner in sustainable agricultural management practices due to its symbiotic relationship with nitrogen-fixing bacteria (Boerema et al., 2016). The reference genome, Williams 82, is ∼1.1 GB with ∼89,500 protein-coding transcripts and 55,589 genes encompassed in 20 chromosomes (Schmutz et al., 2010). The soybean genome is difficult to study due to chromosomal rearrangement, rounds of diploidization, and two major duplication events that occurred 59 and 13 million years ago, making 75% of its genes available in paralogs (Schmutz et al., 2010). Soybean must deal with numerous abiotic stressors, among which are various mineral deficiencies, drought, daylength, and cold weather conditions (Jumrani and Bhatia, 2018). In addition to abiotic stressors which are difficult to control, soybean faces several biotic stressors, including pathogenic stressors such as *Fusarium virguliforme* (causing sudden death syndrome), *Aphis glycines* (soybean aphid), *Sclerotinia sclerotiorum* (causing sclerotinia stem rot), and *Heterodera glycines* Ichinohe, also known as soybean cyst nematode (SCN) (Bradley et al., 2021).

SCN is one of the most destructive pathogens of soybean, first detected in North America in 1954 in North Carolina (Winstead et al., 1955). SCN attacks soybean roots, thereby creating feeding sites within them called syncytia, and robs nutrients from the plant for its own growth and development (Gheysen and Mitchum, 2011). At the J2 (juvenile) stage, the nematode will live and feed in the syncytia for about 3–4 weeks, until they reach the adult stage. Male adults will leave the roots, while females will continue to feed and grow. At one point, the adult females will push through the roots, releasing pheromones to attract adult males for mating. The still females will then deposit eggs near the root while also keeping some within their body, before hardening into cysts and dying. The cysts, containing viable eggs, are able to remain in the soil for up to 10 years until conditions are favorable for them to emerge and infect more soybean roots (Davis and Tylka, 2000).

At present, there exist two commercially used loci for SCN resistance in soybean, the recessive form of *Rhg1* and the dominant form of *Rhg4* (Concibido et al., 1997; Glover et al., 2004; Kim et al., 2010; Liu et al., 2012). The Rhg1 locus consists of a 31 kb multi-gene segment coding for an *α*-SNAP protein (GmSNAP18), a wound-inducible domain protein (WI12; GmWII2), and an amino acid transporter (AAT; GmAAT). All three were shown to be involved in resistance and were mapped to chromosome 18 (Cook et al., 2012; Liu et al., 2017). The Rhg1 locus has two resistance alleles, *rhg1-a* “Peking-type” and *rhg1-b* “PI88788-type.” The *rhg1-a* allele contains a retrotransposon in the GmSNAP18 protein and has a lower copy number for all three proteins (about three or fewer copies), while the *rhg1-b* allele does not contain a retrotransposon in GmSNAP18 and has a higher copy number for the three proteins (∼4–10 copies). The *rhg1-a* allele requires *Rhg4* for complete resistance, while the *rhg1-b* allele does not. The *Rhg4* gene codes for a cytosolic serine hydroxymethyltransferase (SHMT) protein which confers resistance against SCN (Liu et al., 2012).

Current management strategies against SCN remain challenging as soybean varieties containing resistance alleles at Rhg1 or Rhg4 loci are collapsing as more virulent SCN populations are emerging. Since the human population is expected to reach an all-time high in 2050 and continue growing, the threat that SCN poses to soybean yield is significant, fueling a rise in breeding programs which deal with SCN (Yan and Baidoo, 2018; Shaibu et al., 2020; Nissan et al., 2022). There have been significant advancements in SCN research in the hopes of identifying novel genes involved in resistance, such as fine-mapping studies, methylation, large-scale genomics, transgenics, transcriptomics, and proteomics (Shaibu et al., 2020). There is a lack of knowledge when it comes to Rhg-interacting proteins that trigger the hypersensitive response in soybean, which has been problematic in terms of identifying ways to control SCN.

Protein–protein interactions (PPIs) are critical to cellular functions in living organisms. They participate in many different processes including DNA replication, catalysis of metabolic reactions, DNA transcription, suppression or activation of a protein, and transportation of molecules (Peng et al., 2017). Studying PPIs allows for molecular machinery in cells to be identified (De Las Rivas and Fontanillo, 2012). This is possible because proteins often form complex structures to perform specific functions in an interaction network called the “interactome” instead of functioning as individual units (Cusick et al., 2005). Studying PPI networks has aided in identifying gene function (Zhao et al., 2016; Samanfar et al., 2017; Gligorijević et al., 2018), diseases/allergens (Xu and Li, 2006; Dick et al., 2021a), and pharmaceutical discoveries (Yıldırım et al., 2007; Schoenrock et al., 2015). Primarily small-scale studies have identified PPIs through yeast-two-hybrid (Y2H) experiments, tandem affinity purification and mass spectrometry (TAP-MS), and co-immunoprecipitation (Co-IP) techniques (Bensimon et al., 2012; Stynen et al., 2012). For example, a large-scale comprehensive PPI has been performed for *Saccharomyces cerevisiae* in a genome consisting of approximately 6,000 genes, using Y2H studies, proteome chips, and a combination of computational and experimental strategies (Uetz et al., 2000; Zhu et al., 2001; Tong et al., 2002). However, limitations begin to arise with the use of wet-laboratory experiments with larger genomes such as soybean, which is composed of 55,589 genes (Torkamaneh et al., 2021). Some of those limitations include labor costs, scale of study, time constraints, and false positive and negative rates (Zhang et al., 2019). Hence, the use of computational predictors of PPIs has become valuable in molecular biology research. These computational approaches supplement and focus the use of wet-laboratory experiments on targeted, high-confidence predictions. In the last decade, there has been an increase in demand for computational tools that can predict a comprehensive interactome, which is the set of all possible pairwise PPIs within or between proteomes. This has become possible due to the emergence of high-performing computer infrastructure and algorithmic optimizations (Dick et al., 2020). The sequence-based PPI prediction methods used in this study exploit information from previously confirmed PPI sets to determine whether two query proteins will physically interact (Li and Ilie, 2017; Dick et al., 2020).

**FIGURE 1:**
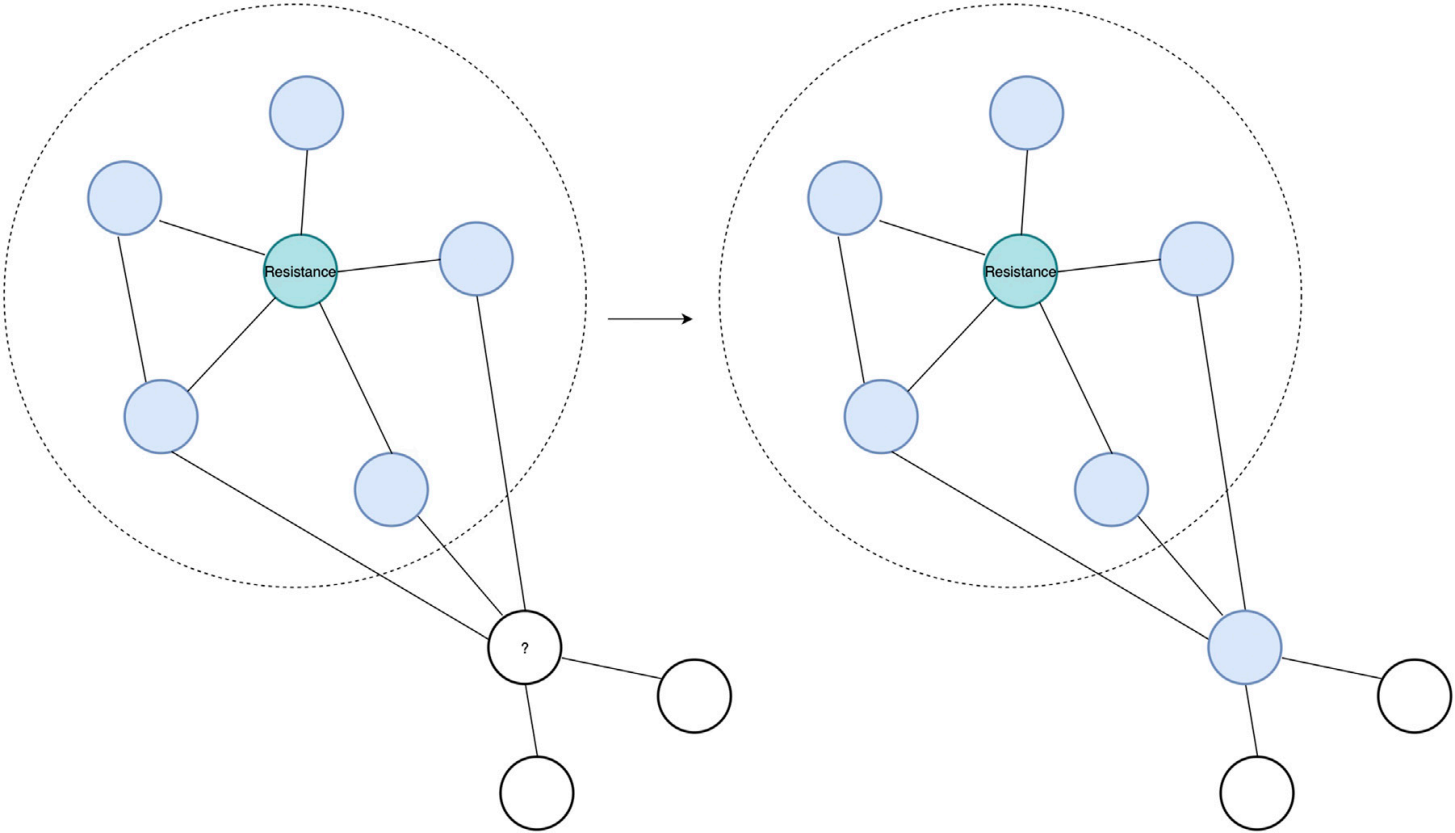
Guilt-by-association approach showing a known SCN resistance protein in teal and its top interacting partners highlighted in blue within the dotted circle. An unknown protein, with a question mark, is observed to interact with the same interacting partner as the resistance protein. Hence, through the “guilt by association” approach, the protein with the question mark can be predicted to interact in the resistance protein pathway due to sharing interacting partners with the known resistance protein.

In this study, we use two complementary PPI prediction methods, the latest version of the Protein–protein Interaction Prediction Engine (PIPE), PIPE4, and the Scoring PRotein INTeractions (SPRINT) predictor, to investigate the soybean proteome (Pitre et al., 2006; Li and Ilie, 2017; Dick et al., 2020). These PPI predictors are applied to predict the PPIs of the entire soybean proteome, which enables studying unannotated proteins through a “guilt by association” approach. Such an approach works on the premise that if an unknown protein is found to be interacting with many proteins exhibiting a given function, there is a heightened chance that the unknown protein also shares that function (see Figure 1) (Rao et al., 2014).

We developed a computational pipeline to identify novel soybean genes possibly involved in the resistance against SCN. Through this analysis, we highlight our most interesting genes by predicting the complete interactome of soybean; we first reveal the direct interactors of Rhg1 and Rhg4 (i.e., the SCN resistance pathway) and, second, discover those unannotated proteins whose interactome overlaps significantly with the pathway.

## 2 Methods

### 2.1 Computational approach, outline, and summary

**FIGURE 2:**
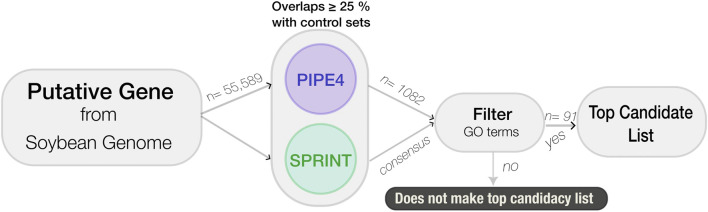
Flowchart of the computational large-scale pipeline used to identity novel soybean genes involved in resistance against SCN.

To identify putative novel genes involved in the resistance pathway to SCN, a data mining pipeline was developed using the longest protein transcript for each of the 55,589 predicted genes. The genes were processed through a sequential cascade of computational analyses. First, PIPE4 and SPRINT were used to predict the entire soybean interactome using the Williams 82 reference genome; decision thresholds are applied to each protein pair within the soybean proteome to predict interactions in the SCN resistance pathway. The resulting candidate list was then refined using GO enrichment of the top candidates using SoyBase’s GO Term Enrichment Tool (https://soybase.org/goslimgraphic_v2/dashboard.php), followed by GO REVIGO (http://revigo.irb.hr) for visualization and further analysis (see Figure 2).

### 2.2 PPI prediction with PIPE4 and SPRINT

Both PIPE4 and SPRINT were used to predict soybean PPIs for all soybean proteins. Due to the lack of experimental soy–soy PPI data, *Arabidopsis thaliana* PPI data were used as a cross-species proxy for training PIPE4 and SPRINT predictors (Dick et al., 2020). This training set consisted of 3,027 *A. thaliana–A. thaliana* confirmed protein interactions between 2,096 proteins. Performing all-to-all soy–soy predictions on 88,647 soy proteins resulted in 3,929,189,628 protein pairs. However, considering only the longest protein sequence isoforms reduces the number of relevant proteins to 56,044 (including Glyma.U proteins), which results in 1,570,492,990 possible interactions. This was performed because both PIPE4 and SPRINT examine the protein sequence, and utilizing the longest sequence will allow the PPI predictors to consider more “windows” for interaction, while removing redundant sequences from the analysis.

**FIGURE 3:**
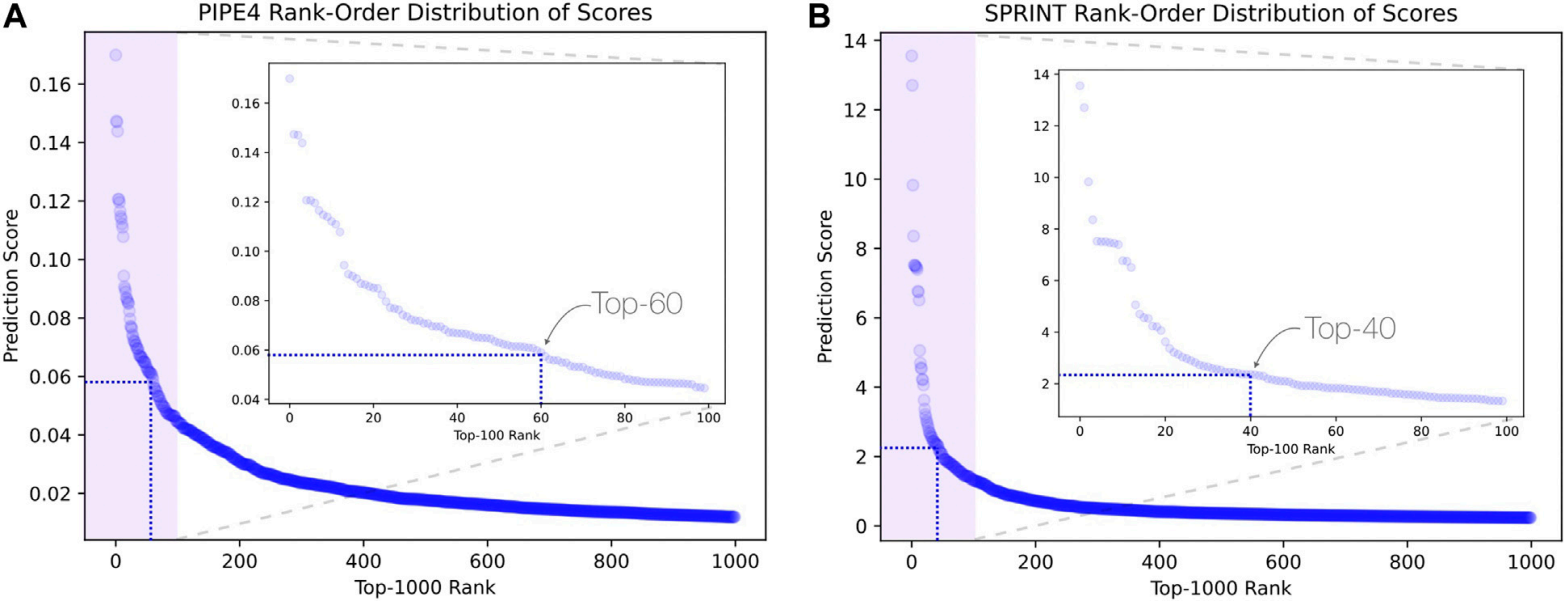
L-shaped curves identifying predicted score plateaus using the top 1,000 predicted interactions for **(A)** PIPE4 and **(B)** SPRINT.

**FIGURE 4:**
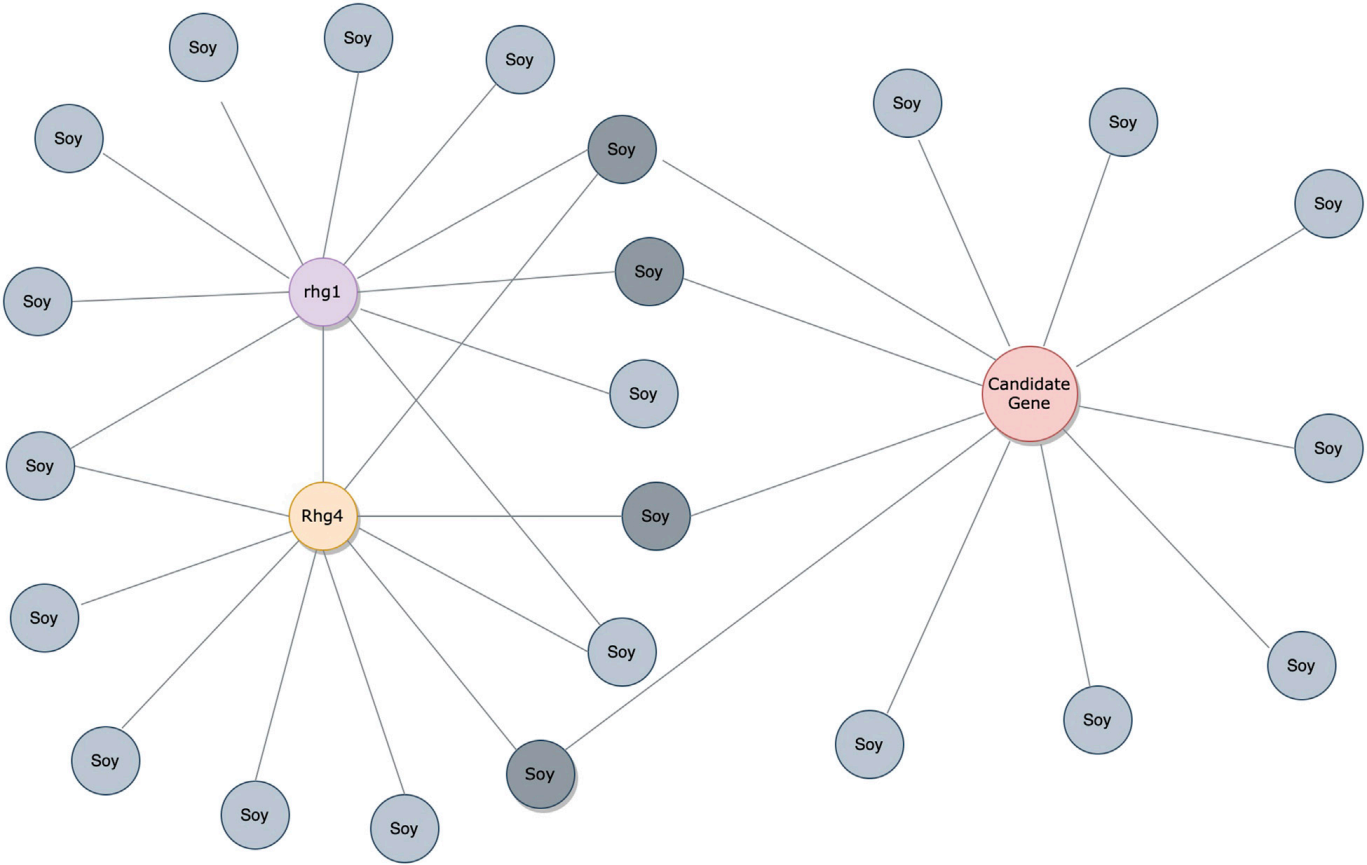
PPI analysis through the guilt-by-association approach. The interactions are denoted by gray soy-interacting protein partners. The red protein is a candidate gene with over 25% overlaps in soy-interacting proteins from positive control sets A^P^/A^S^.

Considering the predictions from both PIPE4 and SPRINT, we determined an overlapping set of highly probable candidate PPIs for subsequent analysis. The top 0.07% of soybean-interacting partners (40 genes) were extracted for each of the 55,589 soy proteins (Glyma.U proteins were used to train PPI predictors but were excluded from our search for novel resistant genes). This highly conservative threshold was determined by plotting the rank-order distribution of the top 1,000 predicted interactions and identifying a “knee” in the L-shaped curve where the predicted score plateaus (Figure 3) as motivated by the following work leveraging a similar methodology (Dick and Green, 2018; Dick et al., 2021a). As shown in Figure 3A, there is a notable step-wise decrease in the PIPE4 prediction scores at rank 60, whereas in Figure 3B, there is a notable step-wise decrease in the SPRINT prediction score at rank 40. For consistency within this work, we selected the highly conservative top 40 cutoff values for both methods. Positive control set A (A^P^) for PIPE4 and (A^S^) for SPRINT comprise the top 40 ranked interacting partners of Rhg1 (*Glyma.18g022400, Glyma.18g022500,* and *Glyma.18g022600*) and Rhg4 (*Glyma.08g108900*) proteins, resulting in 160 PIPE- and 160 SPRINT-identified soybean proteins overlapping with these positive control sets (Supplementary Table S1 and Figure 4). This work follows the “guilt by association” method and was implemented using Python. Any proteins whose top 40 interactors overlapped by at least 25% with the top 40 interactors of the positive control set A^P^ (and A^S^ when repeated using SPRINT) were kept for further analysis. The top 25% were chosen as both PIPE4 and SPRINT predicted the closest numbers of interacting proteins between both predictors at this percentage threshold (Supplementary Figure S1).

Finally, to quantify the predictive performance of both PIPE4 and SPRINT within this cross-species prediction schema, we sought to evaluate the performance of the models on unseen experimentally validated PPI pairs. To this end, we extracted all known soy–soy PPI pairs from BioGRID (https://thebiogrid.org, accessed on 18 May 2023) (*n* = 17) and report the sensitivity of both methods based on the highly conservative top 40 threshold considered in this work (Supplementary Table S2).

### 2.3 Gene Ontology

Soybean genes in the A^P^ and A^S^ positive control sets, as well as the 1,082 candidate list (Supplementary Table S3), were independently run through the SoyBase GO Term Enrichment Tool (https://www.soybase.org/goslimgraphic_v2/dashboard.php, accessed on 17 February 2023) (Grant et al., 2010) to curate the GO term enrichment of each of the two lists (the 58 overlapping proteins in the A^P^/A^S^ list and the 1,082 candidate list). The SoyBase GO:BP output was searched for terms related to “defense,” “response,” and “nematode” to encompass terms of interest identified in the QuickGO database (www.ebi.ac.uk/QuickGO/, accessed on 17 February 2023). Terms of interest in the biological process category included but were not limited to terms involved in defense/resistance such as response to nematode (GO:0009624), response to wounding (GO:0009611), defense response (GO:0006952), response to mechanical stimulus (GO:0009612), response to xenobiotic stimulus (GO:0009410), defense response to bacterium (GO:0042742), jasmonic acid and ethylene-dependent systemic resistance (GO:0009861), response to salicylic acid (GO:0009751), response to ethylene (GO:0009723), response to abscisic acid (GO:0009737), and response to jasmonic acid (GO:0009753). Only defense-related annotations remained in the final lists. The enriched GO:BP terms and *p*-values were then run through REVIGO (http://revigo.irb.hr, accessed on 17 February 2023), a tool used to reduce and visualize large lists of GO terms by scoring mother and daughter ontologies on frequency, relative group size, dispensability, and uniqueness (Supek et al., 2011). REVIGO filtering parameters were set to the medium threshold (0.7), and *A. thaliana* was used as a reference species. REVIGO scatterplots were created using the log_10_ size value of the biological process GO terms across all *A. thaliana* GO terms.

### 2.4 3D structure prediction using AlphaFold2

In this study, we employed the AlphaFold2 algorithm to generate precise 3D structural conformations for the proteins of interest, based on their respective amino acid sequences. AlphaFold2, an advanced deep learning model, utilizes a two-step process to predict protein structures (Jumper et al., 2021). First, it employs a neural network trained on a large database of known protein structures to generate protein-specific potentials. These potentials capture the complex relationships between amino acid sequences and their corresponding structures (Jumper et al., 2021). In the second step, the potentials are utilized to optimize the protein structure by minimizing a predefined energy function. The resulting structures are refined iteratively to achieve higher accuracy (Jumper et al., 2021). By leveraging AlphaFold2, we obtained highly accurate 3D structural conformations for the proteins of interest, facilitating a comprehensive understanding of their molecular functions and interactions. We make this predicted structural information available to the broader research community within the GitHub repository associated with this work (https://github.com/earezza/Soybean-Large-Scale-PPI-Analysis).

## 3 Results

### 3.1 Results for the positive control sets A^P^ and A^S^

PIPE4 and SPRINT predicted the top 40 (or top 0.07%) interacting partners of Rhg1 and Rhg4 proteins (*Glyma.18g022400, Glyma.18g022500, Glyma.18g022600*, and *Glyma.08g108900*), resulting in the A^P^ and A^S^ positive control sets (Supplementary Table S1) resulting in 58 overlapping proteins.

### 3.2 Gene Ontology results for the overlapping proteins in positive control sets A^P^ and A^S^

**FIGURE 5:**
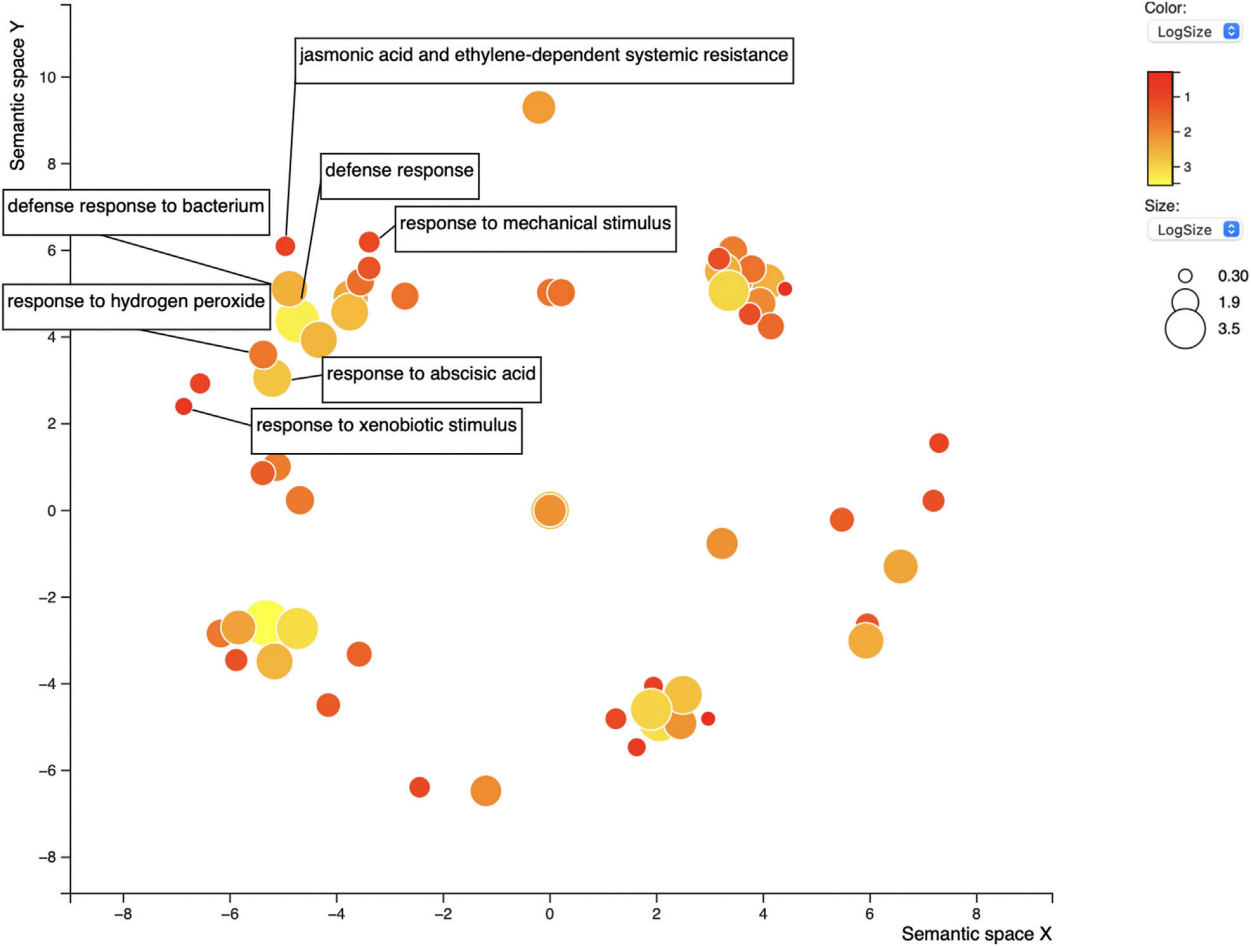
REVIGO scatterplot of the 86 GO terms for the 58 overlapping proteins found in positive control lists A^P^/A^S^. GO search was performed using medium 0.7 list size and using *Arabidopsis thaliana* as the species to work with. The size and color of the circles represent logSize value; higher logSize values indicate high numbers of a term and/or its daughter terms within the total database for *A. thaliana*; terms that are highly represented have larger bubbles.

For the 58 genes found to overlap between both sequence-predictors, we used Gene Ontology (GO) to better understand their roles in relation to SCN resistance or defense response, as well as help clarify the ontologies of the Rhg1 and Rhg4 interactome. SoyBase’s GO term enrichment analysis of the overlapping predicted interacting protein list of genes identified 86 biological process (BP) and molecular function (MF) terms strongly associated with these genes (Supplementary Table S4). The GO analysis was used to search for defense-related annotations. Only those candidate proteins that were strongly associated with defense-related GO terms were retained, resulting in 19 out of the 58 genes. The SoyBase GO terms did not include any hits for “nematode;” however, other defense-related terms were found. Subsequent analysis using REVIGO reduced the GO terms to 68 from 86 (see Figure 5). The terms “defense response” (GO:0006952) and “response to mechanical stimulus” (GO:0009612) were two terms listed with 0.346 and 0.309 dispensability values, respectively (Supplementary Table S5). From the SoyBase GO enrichment data, it was evident that five genes were responsible for both terms (Table 1). In addition to these valuable GO terms, there were other defense-related terms present within the control list including but not limited to “response to xenobiotic stimulus” (GO:0009410) with a dispensability score of 0. Another enriched GO term found in the overlapping proteins in the control list is “defense response to bacterium” (GO:0042742), which contains a dispensability score of 0.488.

**TABLE 1:** Top 19 defense-related interacting partners of Rhg1 and Rhg4 proteins predicted by both PIPE4 and SPRINT engines and their corresponding defense-related GO terms.

Genes in both A^P^ and A^S^ lists	GO terms	GO term ID	TAIR10 hit
*Glyma.08G032900*			Heat shock protein 81–2
*Glyma.17G182500*	Defense response	GO:0006952	Heat shock protein 81–2
*Glyma.17G220000*			Heat shock protein 81–2
*Glyma.19G098200*	Response to mechanical stimulus	GO:0009612	Heat shock protein 81–2
*Glyma.20G037900*			Heat shock protein 81–2
*Glyma.04G200500*			Basic helix–loop–helix (bHLH) DNA-binding superfamily protein
*Glyma.06G165000*			Basic helix–loop–helix (bHLH) DNA-binding superfamily protein
*Glyma.08G008200*	Response to xenobiotic stimulus	GO:0009410	Basic helix–loop–helix (bHLH) DNA-binding superfamily protein
*Glyma.16G049400*			Basic helix–loop–helix (bHLH) DNA-binding superfamily protein
*Glyma.16G147200*			Basic helix–loop–helix (bHLH) DNA-binding superfamily protein
*Glyma.19G102000*			Basic helix–loop–helix (bHLH) DNA-binding superfamily protein
*Glyma.09G131500*	Response to bacterium	GO:0042742	Heat shock protein 90.1
*Glyma.16G178800*			Heat shock protein 90.1
*Glyma.01G245100*	Jasmonic acid and	GO:0009861	Histone deacetylase 1
*Glyma.04G000200*	ethylene-dependent		Histone deacetylase 1
*Glyma.06G000100*	systemic resistance		Histone deacetylase 1
*Glyma.11G000300*			Histone deacetylase 1
*Glyma.04G187000*	Response to abscisic acid	GO:0009737	Histone deacetylase 6
*Glyma.05G040600*			Histone deacetylase 6
*Glyma.06G178800*			Histone deacetylase 6
*Glyma.17G085700*			Histone deacetylase 6

In addition to the typical defense GO terms, other ontologies related to hormone response that also play a role in plant defense were present, such as “jasmonic acid and ethylene-dependent systemic resistance” (GO:0009861, dispensability: 0.117) and “response to abscisic acid” with four genes (GO:0009737, dispensability: 0.510) (Table 1).

### 3.3 Interactions with the positive control sets A^P^ and A^S^

PIPE4 predicted 5,763 genes with 25% or more overlaps in soybean-interacting partners with positive control set A^P^, while SPRINT predicted 6,153 genes with at least 25% overlaps in soybean-interacting partners with positive control set A^S^. Comparing PIPE4 and SPRINT results showed that 1,086 genes were common to both with the top four being *Rhg1* and *Rhg4* genes themselves (Supplementary Table S3).

**FIGURE 6:**
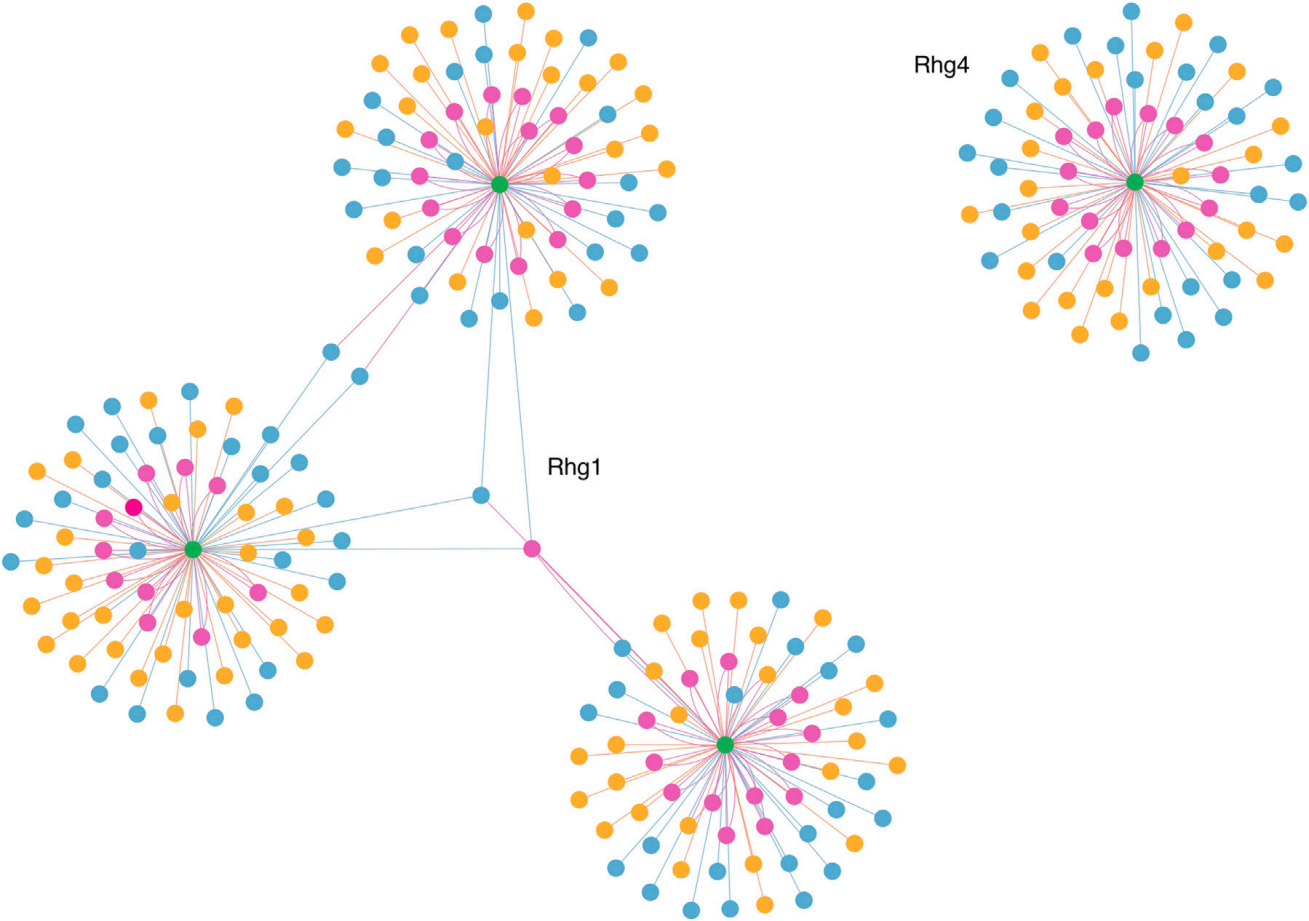
Network-based depiction of the Rhg1 and Rhg4 interaction partners by both PIPE4 and SPRINT, as well as their overlapping sets. Green nodes are the Rhg1 and Rhg4 proteins, yellow nodes are predicted by PIPE4, blue nodes are predicted by SPRINT, and pink nodes represent the overlapping predictions. Link for the interactive plot: https://cu-bic.ca/soybean-rgh1-rgh4/.

To visualize and interpret the predicted *Rhg1* and *Rhg4* partners using PIPE4 and SPRINT, Figure 6 shows a network-based representation that highlights the overlap for both sets: green nodes are the Rhg1 and Rhg4 proteins, yellow nodes are predicted by PIPE4, blue nodes are predicted by SPRINT, and pink nodes represent the overlapping predictions. To better interpret those proteins and their relationships within this network, a fully interactive variant of this network is available at the following link: https://cu-bic.ca/soybean-rgh1-rgh4/.

### 3.4 Gene Ontology analysis for top candidate genes

**FIGURE 7:**
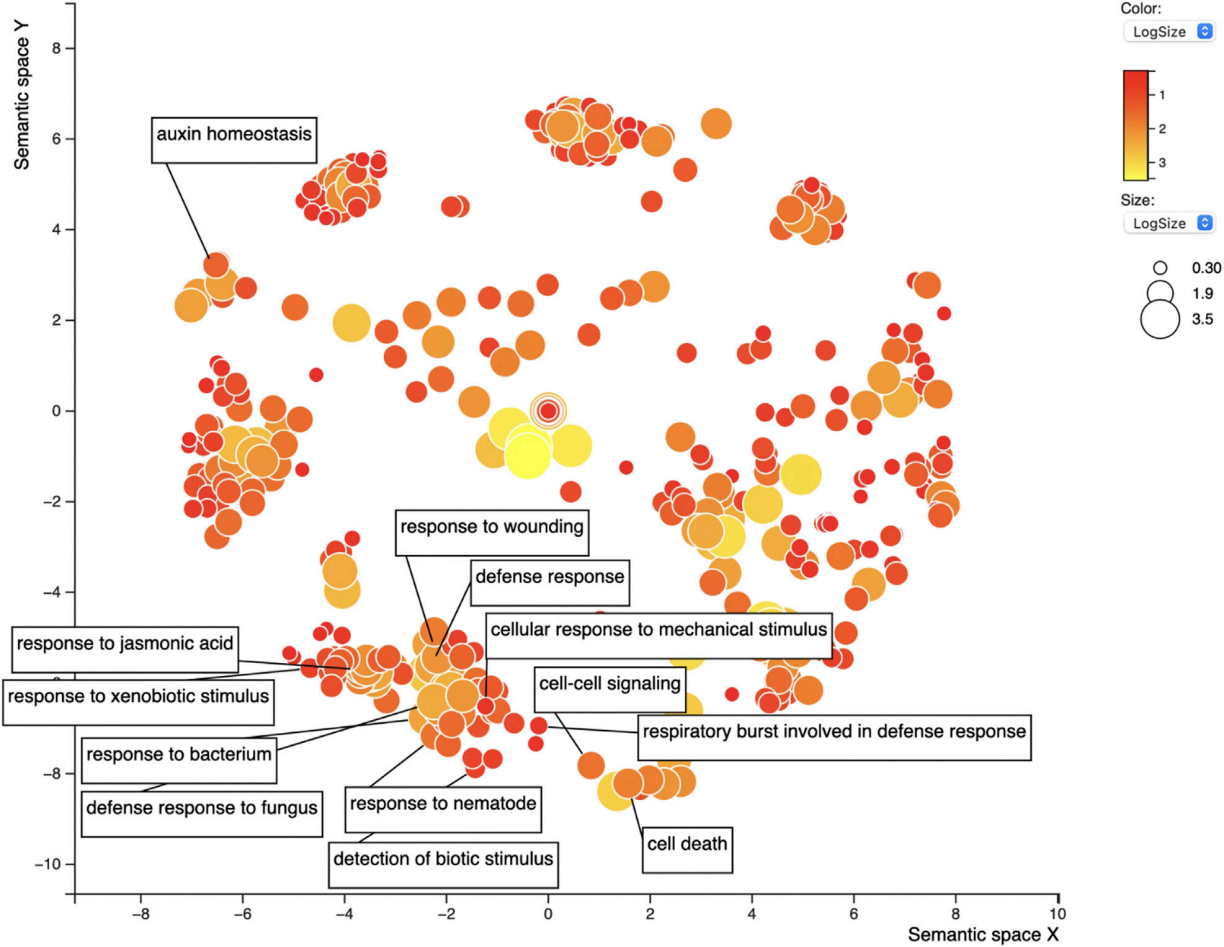
REVIGO scatterplot of the 1,183 GO terms for the top 1,082 candidate soybean genes for resistance against SCN. GO search was performed using medium 0.7 list size and using *Arabidopsis thaliana* as the species to work with. The size and color of the circles represent logSize value; higher logSize values indicate high numbers of a term and/or its daughter terms within the total database for *A. thaliana*; terms that are highly represented have larger bubbles.

The set of 1,086 genes (1,082 after excluding the *Rhg1* and *Rhg4* genes themselves) was assessed for GO term enrichment to refine the search for genes related to SCN infection or defense response. The gene IDs were input into the SoyBase GO Term Enrichment Tool, and the GO enrichment data were extracted. The GO enrichment identified 1,183 unique GO terms from this gene list (Supplementary Table S6). The term “response to nematode” (GO:0009624, dispensability: 0.921) was overrepresented in the GO output; five genes were responsible for this enrichment. Also, among the SoyBase GO term enrichment output, “regulation of nematode larval development” (GO:0061062) was overrepresented among the data and also included one of five genes from the previous GO term. REVIGO filtering reduced the list of GO terms by 30%, resulting in 765 GO terms. The “response to nematode” term was retained after filtering; this is a daughter term of “defense response” (GO:0006952), which was the 15th most frequent GO term (Supplementary Table S7; Figure 7). Of the list of genes, 19 were responsible for the “defense response” term (dispensability: 0). Similarly, “regulation of defense response” (GO:0031347, dispensability: 0.932) was identified and made up a list of six genes, “response to xenobiotic stimulus” (GO:0009410, dispensability: 0.936) was also enriched in six genes, “response to wounding” (GO:0009611, dispensability: 0.922) in 23 genes, “response to mechanical stimulus” (GO:0009612, dispensability: 0.915) three genes, and “innate to immune response” (GO:0045087, dispensability: 0.899) is another important term related to resistance and defense-related genes with three genes being responsible. Finally, “detection of biotic stimulus” (GO:0009595, dispensability: 0.939) is the last GO term of interest overrepresented in this list with eight genes.

In addition to defense-related GO terms, GO terms related to hormones responsible for defense were also identified within this list including “response to jasmonic acid” (GO:0009753, dispensability: 0.899) enriched in nine genes, “response to ethylene” (GO:0009723, dispensability: 0.904) in 11 genes, “response to salicylic acid” (GO:0009751, dispensability: 0.901) in seven genes, and finally, “response to abscisic acid” (GO:0009737, dispensability: 0.890) enriched in 28 genes. All genes are presented in Table 2.

**TABLE 2:** Top 91 candidate soybean genes for resistance against SCN identified from the genome-wide computational analysis from the 1,082 gene list and their corresponding defense-related GO terms.

Genes in both A^P^ and A^S^ lists	GO terms	GO term ID	TAIR10 hit
*Glyma.08G120500*	Response to nematode	GO:0009624	Major facilitator superfamily protein
*Glyma.08G265700*			Growth-regulating factor 1
*Glyma.11G228300*			Transmembrane amino acid transporter family protein
*Glyma.17G152300*			Purine permease 10
*Glyma.18G029000*			Transmembrane amino acid transporter family protein
*Glyma.08G265700*	Regulation of nematode larval development	GO:0061062	Growth-regulating factor 1
*Glyma.01G013100*	Defense response	GO:0006952	NB-ARC domain-containing disease resistance protein
*Glyma.01G030100*			NB-ARC domain-containing disease resistance protein
*Glyma.01G149200*			NB-ARC domain-containing disease resistance protein
*Glyma.02G020300*			WRKY DNA-binding protein 72
*Glyma.02G051200*			Disease resistance protein (TIR-NBS-LRR class)
*Glyma.04G035000*			Allene oxide synthase
*Glyma.04G068000*			Overexpressor of cationic peroxidase 3
*Glyma.06G259100*			Disease resistance protein (TIR-NBS-LRR class), putative
*Glyma.06G310000*			Disease resistance protein (TIR-NBS-LRR class) family
*Glyma.07G153500*			Receptor-like protein 27
*Glyma.09G090400*			NB-ARC domain-containing disease resistance protein
*Glyma.09G102400*			MLP-like protein 34
*Glyma.11G131300*			Leucine-rich repeat (LRR) family protein
*Glyma.12G055500*			Leucine-rich repeat (LRR) family protein
*Glyma.14G078600*			Allene oxide synthase
*Glyma.15G209200*			Polygalacturonase inhibiting protein 1
*Glyma.16G134000*			S-adenosyl-L-methionine-dependent methyltransferase superfamily protein
*Glyma.18G263900*			Cyclic nucleotide-regulated ion channel family protein
*Glyma.19G055000*			Disease resistance protein (TIR-NBS-LRR class) family
*Glyma.02G105900*	Regulation of defense response	GO:0031347	TEOSINTE BRANCHED, cycloidea and PCF (TCP) 14
*Glyma.02G187900*			Protein kinase superfamily protein
*Glyma.10G271400*			Protein kinase superfamily protein
*Glyma.16G064100*			Leucine-rich repeat receptor-like protein kinase family protein
*Glyma.16G064200*			Leucine-rich repeat receptor-like protein kinase family protein
*Glyma.19G030900*			Plastid transcription factor 1
*Glyma.02G082800*	Response to xenobiotic	GO:0009410	VIRE2-interacting protein 1
*Glyma.07G161500*	stimulus		Tetratricopeptide repeat (TPR)-like superfamily protein
*Glyma.10G172500*			RING/FYVE/PHD zinc-finger superfamily protein
*Glyma.12G027700*			Tetratricopeptide repeat (TPR)-containing protein
*Glyma.17G007300*			Ferredoxin hydrogenases
*Glyma.20G217700*			RING/FYVE/PHD zinc-finger superfamily protein
*Glyma.02G103500*	Response to wounding	GO:0009611	S-adenosyl-L-methionine-dependent methyltransferase superfamily protein
*Glyma.02G113600*			Chitinase A
*Glyma.04G007700*			Arginine decarboxylase 2
*Glyma.04G035000*			Allene oxide synthase
*Glyma.05G196100*			Diacylglycerol kinase 2
*Glyma.06G037000*			Protein of unknown function
*Glyma.06G160500*			Myb domain protein 4
*Glyma.06G186200*			Unknown protein
*Glyma.07G004700*			Proline extension-like receptor kinase 1
*Glyma.07G048700*			O-methyltransferase 1
*Glyma.08G071000*			White–brown complex homolog protein 11
*Glyma.08G338900*			UDP-glycosyltransferase superfamily protein
*Glyma.09G160000*			White–brown complex homolog protein 11
*Glyma.09G162400*			UDP-glucosyl transferase 71B6
*Glyma.10G010400*			Myb domain protein 2
*Glyma.10G180800*			Myb domain protein 15
*Glyma.12G191400*			Hydroperoxide lyase 1
*Glyma.12G194200*			Glutamate receptor 3.4
*Glyma.13G248800*			S-locus lectin protein kinase family protein
*Glyma.14G078600*			Allene oxide synthase
*Glyma.15G080300*			HXXXD-type acyl-transferase family protein
*Glyma.16G134000*			S-adenosyl-L-methionine-dependent methyltransferase superfamily protein
*Glyma.16G209400*			White–brown complex homolog protein 11
*Glyma.06G037000*	Response to mechanical	GO:0009612	Protein of unknown function
*Glyma.12G184500*	stimulus		bZIP transcription factor family protein
*Glyma.13G316900*			bZIP transcription factor family protein
*Glyma.13G161700*	Innate to immune	GO:0045087	Calmodulin-binding receptor-like cytoplasmic kinase 3
*Glyma.13G323400*	response		Phosphatidate cytidylyltransferase family protein
*Glyma.18G294800*			Protein kinase family protein
*Glyma.02G176300*	Detection of biotic stimulus	GO:0009595	Phytochelatin synthase 1 (PCS1)
*Glyma.04G035000*			Allene oxide synthase
*Glyma.05G007100*			Carbonic anhydrase 1
*Glyma.05G151000*			Subtilase family protein
*Glyma.09G066600*			MAP kinase kinase 2
*Glyma.14G078600*			Allene oxide synthase
*Glyma.18G208800*			WRKY DNA-binding protein 33
*Glyma.19G007700*			Carbonic anhydrase 1
*Glyma.04G007700*			Arginine decarboxylase 2
*Glyma.04G035000*			Allene oxide synthase
*Glyma.06G160500*	Response to jasmonic acid	GO:0009753	Myb domain protein 4
*Glyma.10G010400*			Myb domain protein 2
*Glyma.10G180800*			Myb domain protein 15
*Glyma.14G078600*			Allene oxide synthase
*Glyma.16G134000*			S-adenosyl-L-methionine-dependent methyltransferases superfamily protein
*Glyma.17G076100*			Glycosyl hydrolase family protein with chitinase insertion domain
*Glyma.19G030900*			Plastid transcription factor 1
*Glyma.02G080200*	Response to ethylene	GO:0009723	Integrase-type DNA-binding superfamily protein
*Glyma.04G007700*			Arginine decarboxylase 2
*Glyma.07G175000*			Anthranilate synthase beta subunit 1
*Glyma.09G162400*			UDP-glucosyl transferase 71B6
*Glyma.10G010400*			Myb domain protein 2
*Glyma.10G180800*			Myb domain protein 15
*Glyma.11G228300*			Transmembrane amino acid transporter family protein
*Glyma.12G059000*			Metal tolerance protein B1
*Glyma.12G225600*			MATE efflux family protein
*Glyma.18G026700*			CRINKLY4-related 3
*Glyma.18G029000*			Transmembrane amino acid transporter family protein
*Glyma.03G214100*	Response to salicylic acid	GO:0009751	Domain-containing protein
*Glyma.04G101900*			Myb domain protein 93
*Glyma.06G103300*			Myb domain protein 93
*Glyma.06G160500*			Myb domain protein 4
*Glyma.10G010400*			Myb domain protein 2
*Glyma.13G070900*			Peroxidase superfamily protein
*Glyma.19G011700*			Peroxidase superfamily protein
*Glyma.02G105900*	Response to abscisic acid	GO:0009737	TEOSINTE BRANCHED, cycloidea and PCF (TCP) 14
*Glyma.04G007700*			Arginine decarboxylase 2
*Glyma.04G057000*			Copper transporter 5
*Glyma.04G068000*			Overexpressor of cationic peroxidase 3
*Glyma.04G101900*			Myb domain protein 93
*Glyma.06G032600*			GYF domain-containing protein
*Glyma.06G103300*			Myb domain protein 93
*Glyma.07G003000*			Galactose mutarotase-like superfamily protein
*Glyma.08G071000*			White–brown complex homolog protein 11
*Glyma.08G223600*			Galactose mutarotase-like superfamily protein
*Glyma.08G265700*			Growth-regulating factor 1
*Glyma.09G057300*			Galactose mutarotase-like superfamily protein
*Glyma.09G160000*			White–brown complex homolog protein 11
*Glyma.09G162400*			UDP-glucosyl transferase 71B6
*Glyma.09G171100*			Homeodomain-like superfamily protein
*Glyma.10G010400*			Myb domain protein 2
*Glyma.10G180800*			Myb domain protein 15
*Glyma.12G022500*			Unknown
*Glyma.12G181400*			Histone deacetylase 2C
*Glyma.13G070900*			Peroxidase superfamily protein
*Glyma.13G329700*			Related to AP2.7
*Glyma.14G140900*			BURP domain-containing protein
*Glyma.15G163600*			Galactose mutarotase-like superfamily protein
*Glyma.16G209400*			White–brown complex homolog protein 11
*Glyma.17G076100*			Glycosyl hydrolase family protein
*Glyma.17G249900*			GYF domain-containing protein
*Glyma.19G011700*			Peroxidase superfamily protein
*Glyma.20G137200*			Cysteine-rich RLK (RECEPTOR-like protein kinase)

## 4 Discussion

In this research, we used various bioinformatics tools including two machine learning PPI predictors, PIPE4 and SPRINT, to scan the entire soybean genome in search of novel genes involved in resistance against SCN (Li and Ilie, 2017; Dick et al., 2020). We predicted the soybean interactome using *Arabidopsis thaliana* as a proxy training species, due to the lack of confirmed PPIs in the organism in question. The requirement to use a proxy species will somewhat reduce the accuracy of these methods (Dick et al., 2020). Therefore, we used a highly conservative score threshold, retaining only the top 0.07% of predicted interactions for each protein (i.e., top 40 predictions). Using predictions common to both PIPE4 and SPRINT also provided a conservative approach. The subsequent stages of the analysis pipeline (i.e., threshold filtration and REVIGO filtration) were designed to further filter the list of potential interactors, thereby increasing our confidence in the final list of candidate genes hypothesized to be associated with SCN resistance in soybean.

Early comparisons of the performance of SPRINT to the ancestral PIPE2 algorithm list SPRINT performance as having a sensitivity of 12.92% at a 99.95% specificity compared to PIPE2 having a sensitivity of 6.57% at a 99.95% specificity (Li and Ilie, 2017). In this work, we quantified the performance of the PIPE4 and SPRINT methods using known (experimentally validated physical interactions) *G. max*-*G.* max extracted from BioGRID: 17 unseen PPI pairs not used in the training of either method that can be used to quantify the performance of the two methods. From the highly conservative top 40 predictions considered in this work, PIPE4 detected 2/17 pairs, achieving a sensitivity of 11.8%, while SPIRNT detected 4/17 pairs, achieving a sensitivity of 23.5% (Supplementary Table S2). Given the severely limited availability of experimentally validated PPIs (only *n* = 17 physical interactions), these estimates are extremely conservative and may under-report the performance of both PIPE4 and SPRINT. Numerous recent comparisons of the SPRINT algorithm to the latest PIPE4 algorithm indicate that both methods perform similarly and complement one another in massive-scale interactome studies (Dick et al., 2020; Dick et al., 2021b). Importantly, both methods are tuned to be highly conservative and aim to minimize the false positive rate, making them applicable to interactome-scale screening analysis. At present, PIPE4 and SPRINT predictive performance approaches that of wet-laboratory studies such as tap-tagging and yeast-two-hybrid studies, but on a much faster and larger scale and with lower running costs (Pitre et al., 2006; Li and Ilie, 2017). These two PPI predictors function by using a sequence-based approach utilizing the primary sequence of amino acids and a known dataset of interacting partners. They differ primarily in how short regions of sequence similarity are defined when comparing query proteins to known PPIs. They are both algorithms that automatically learn and extract sequence patterns important to PPIs, learned directly from the training examples of known PPIs (Li and Ilie, 2017; Dick et al., 2020). Since we have confirmed soybean proteins that play a major role in resistance against SCN, Rhg1 and Rhg4, we were able to use PIPE4 and SPRINT to extract the top-ranked predicted interacting soy partners of Rhg1 and Rhg4 to act as positive control groups. By comparing top-ranked interacting partners between these positive control proteins and all other soybean proteins, we can identify candidate soybean proteins that are likely to share resistance-related function with Rhg1 and Rhg4 through the guilt-by-association approach.

To this point in time, researchers have struggled to identify genes involved in the host–pathogen relationship between soybean and SCN as the defense mechanism of soybean against SCN seems to be different from the typical “R gene” type of resistance. This can be seen in the discovery of *Rhg1* and *Rhg4* genes (Cook et al., 2012; Liu et al., 2012; Liu et al., 2017), as well as the later discovery of a pathogenesis-related protein GmPR08-Bet VI (*Glyma.08g230500*) as an interacting partner (Lakhssassi et al., 2020)*.* Hence, we posed the question “Can we predict the top interacting partners for Rhg1 and Rhg4 with a high degree of accuracy through a computational large-scale approach, and if so, what kind of genes will we find to be present within that relationship?” To the best of our knowledge, this is the first study to attempt to answer this question on a large scale. By tackling this problem and making our data available for scientists, we can open possibilities for further research on this relationship.

By filtering and visualizing the GO terms of A^P^/A^S^ positive control lists by first using the SoyBase GO Term Enrichment Tool and then using REVIGO, we identified that our two PPI predictors, PIPE4 and SPRINT, made overlapping predictions of Rhg1 and Rhg4 top interacting proteins with GO terms involved in defense response (GO:0006952) and response to mechanical stimulus (GO:0009612). Seven genes were responsible for these enriched functions (*Glyma.17G182500, Glyma.08G032900, Glyma.20G037900, Glyma.17G220000, Glyma.10G098300, Glyma.19G098200,* and *Glyma.03G114400*). As shown in Table 1, our two PPI predictors made common predictions for proteins that interact with Rhg1 and Rhg4 in the broad “defense response” category, also in additional defense-related GO terms including but not limited to response to xenobiotic stimulus (GO:0009410), defense response to bacterium (GO:0042742), and jasmonic acid and ethylene-dependent systemic resistance (GO:0009861) (Table 1). We note here that the genes predicted using both engines have a higher chance of being true predictions and that we may now be one step closer to understanding the soybean–SCN relationship.

The major resistance gene at *Rhg1* is an alpha-soluble N-ethylmaleimide-sensitive factor (NSF) attachment protein (α-SNAP) that is present in multiple copies in resistant lines (Cook et al., 2012). Normally, this vesicular trafficking chaperone binds SNARE complexes and stimulates their disassembly by activating NSF. However, the resistance allele is defective in interacting with NSF, and the overexpression of *α-SNAP* in the syncytium leads to the disruption of vesicle trafficking and cytotoxic levels of NSF (Bayless et al., 2016). In our study, 15 proteins were predicted to interact with *α-SNAP* by both predictors. Many of these were protein kinases. It was shown that mitogen-activated protein kinases were overexpressed in the syncytium, play important signal transduction and membrane trafficking roles, and may be involved in the defense response to nematode infection (McNeece et al., 2019). The second gene at *Rhg1*, AAT_Rhg1_, is a putative amino acid transporter. It was recently shown that AAT_Rhg1_ accumulates along the path of nematode invasion and physically interacts with NADPH oxidase (Han et al., 2023). This results in significant reactive oxygen species (ROS) increase in resistant lines. Most of the 11 proteins predicted to interact with AAT_Rhg1_, by both predictors in the present work, are heat shock proteins. These proteins are central to the oxidative stress responses and may be partnering with AAT_Rhg1_ in SCN resistance. The other main SCN resistance quantitative trait locus (QTL) in soybean, *Rhg4*, encodes a serine hydroxymethyltransferase (SHMT) (Liu et al., 2012). Our predictions revealed that most of the 15 interactions predicted by both predictors are with proteins involved in the ubiquitin-dependent protein catabolic process. While the mechanism of resistance involving SHMT_Rhg4_ is still not fully understood, the ubiquitin proteasome system is involved in host-defense in many different pathosystems (Kud et al., 2019).

In addition to investigating the positive control sets A^P^/A^S^, we were also interested in identifying additional proteins involved in the resistance pathway against SCN. Hence, we posed another question: “If we can predict interacting partners of known resistance genes, can we use those predictions with a guilt-by-association approach to identify novel genes involved in the resistance pathway by scanning the PPI network of the entire soybean proteome?” We wanted to do this on a large scale as current resistant varieties are becoming increasingly susceptible to the pest (Kofsky et al., 2021). We wanted to identify additional genes, through a computational approach, for the possibility of stacking resistance. We identified 1,082 candidates from the entire soybean genome based on the level of overlaps between their interacting partners and the top interacting partners of Rhg1 and Rhg4. Interestingly, by filtering the enriched GO terms, we identified five genes with ontologies related to response to nematode, *Glyma.18G029000*, *Glyma.11G228300*, *Glyma.08G120500*, *Glyma.17G152300*, and *Glyma.08G265700*, or regulation to larval development (GO:0061062) (*Glyma.08G265700*). These offer good targets for future validation studies to characterize their role in resistance against SCN. Among the 1,082 genes, 91 were highlighted (Table 2) based on predicted functions that could be compatible with resistance and will warrant future research, for example, *Glyma.19G055000* is a *toll-interleukin-1 receptor, nucleotide-binding site,* and *leucine-rich repeat* (TIR-NBS-LRR) disease resistance protein. This class includes many classical plant disease resistance genes. Furthermore, through literature search, it was identified that five out of the top predictions from Tables 1 and 2 had genes present within a ± 50 kb window of recent QTLs and genome-wide association studies, i.e., *Glyma.04g007700* (Li et al., 2016), *Glyma.06g186200* (Li et al., 2016), *Glyma.10g172500* (Tran et al., 2019), *Glyma.17g085700* (Li et al., 2016), and *Glyma.18g029000* (Chang et al., 2017). Interestingly, two other genes were found within the QTL regions, SCN-2 (*Glyma.08g223600*) and SCN-3 (*Glyma.08g338900*) (Swaminathan et al., 2018).

**FIGURE 8:**
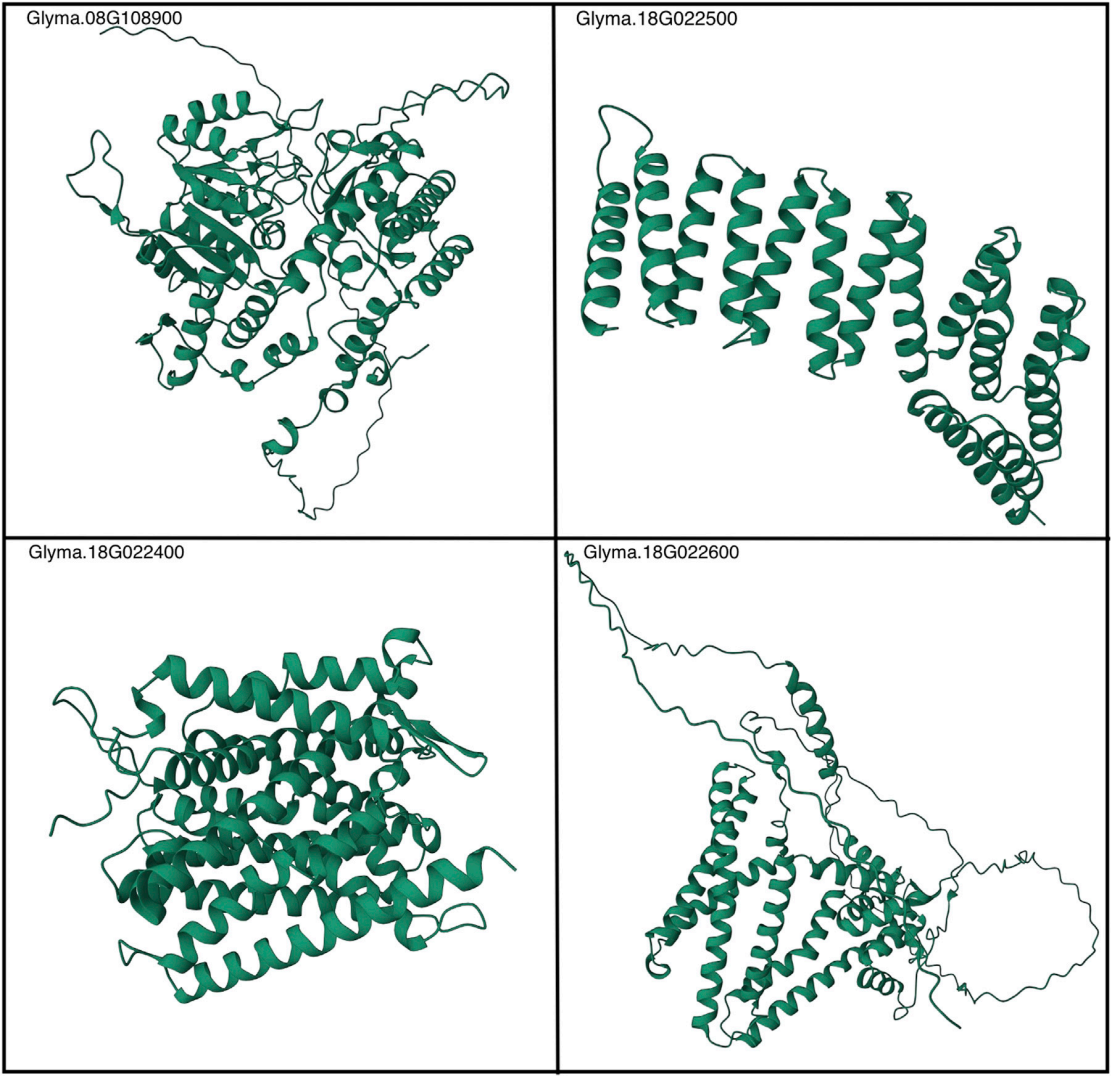
AlphaFold2-generated depictions of a static view of Rhg1 and Rhg4 folded proteins. See the following link for more details: https://github.com/earezza/Soybean-Large-Scale-PPI-Analysis.

Finally, the predicted structures generated by AlphaFold2 offer significant utility to the broader research community, both in the extension of the research findings herein and more broadly in the realm of host–pathogen biology. These highly accurate 3D structural conformations, available at https://github.com/earezza/Soybean-Large-Scale-PPI-Analysis, serve as valuable resources for scientists investigating the molecular mechanisms underlying plant defense mechanisms. Figure 8 depicts a static view of the folded proteins, and the proteins most relevant within this work and additional structures are given in Supplementary Table S2. By incorporating the predicted structures into their research, scientists can gain insights into putative PPIs, candidate ligand-binding sites, and potential enzymatic activities, facilitating the development of strategies to enhance plant resistance against pathogens. The predicted structures also provide a starting point for experimental studies, allowing for validation and refinement through techniques like X-ray crystallography and cryo-electron microscopy. Overall, the use of AlphaFold2 predictions holds significant promise for advancing our understanding of host–pathogen interactions and contributing to the development of innovative approaches in plant biology. Given the sparsity of known PPIs and/or experimentally determined protein structures within the *G. max*-*G.* max proteome, it is our recommendation that subsequent research initiatives leverage these state-of-the-art AI methods to increasingly expand their representation within large-scale consortium databases such as the AlphaFold protein structure database (Varadi et al., 2021).

## 5 Conclusion

In this paper, we provide an approach to scan the entire soybean proteome and use a guilt-by-association method, in addition to a multistep workflow, to predict the most likely novel candidates involved in resistance against SCN. This pipeline combined two machine learning tools, PIPE4 and SPRINT, and illustrated the potential of new technological advances to facilitate gene discovery. We believe that these tools can be used to predict other resistance protein-interacting partners and will allow scientists to focus their research in a much more efficient manner to address existing and emergent diseases.

## Data Availability

The original contributions presented in the study are included in the article/Supplementary Material; further inquiries can be directed to the corresponding author.
