# Supplementary figures and images for: Large-scale data mining pipeline for identifying novel soybean genes involved in resistance against the soybean cyst nematode
Source: Front Bioinform. 2023 Jun 20;3:1199675. doi: 10.3389/fbinf.2023.1199675 (PMC10319130; doi:10.3389/fbinf.2023.1199675)

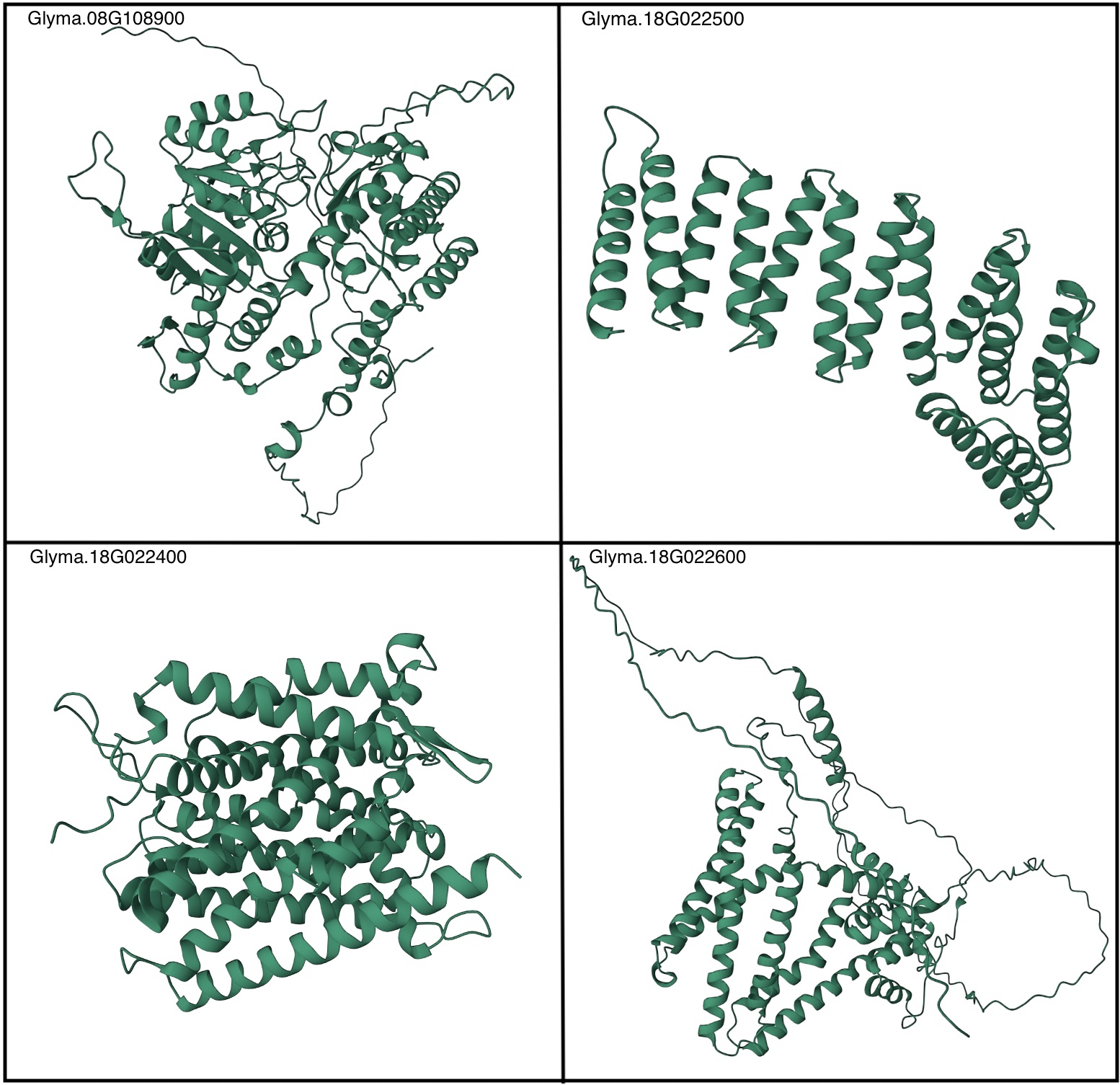

Supplement: Supplementary file 1 [file Image1.JPEG]
