# Supplementary material for: Large-scale data mining pipeline for identifying novel soybean genes involved in resistance against the soybean cyst nematode
Source: Front Bioinform. 2023 Jun 20;3:1199675. doi: 10.3389/fbinf.2023.1199675 (PMC10319130; doi:10.3389/fbinf.2023.1199675)
Supplement: Supplementary file 2 [file DataSheet1.PDF]

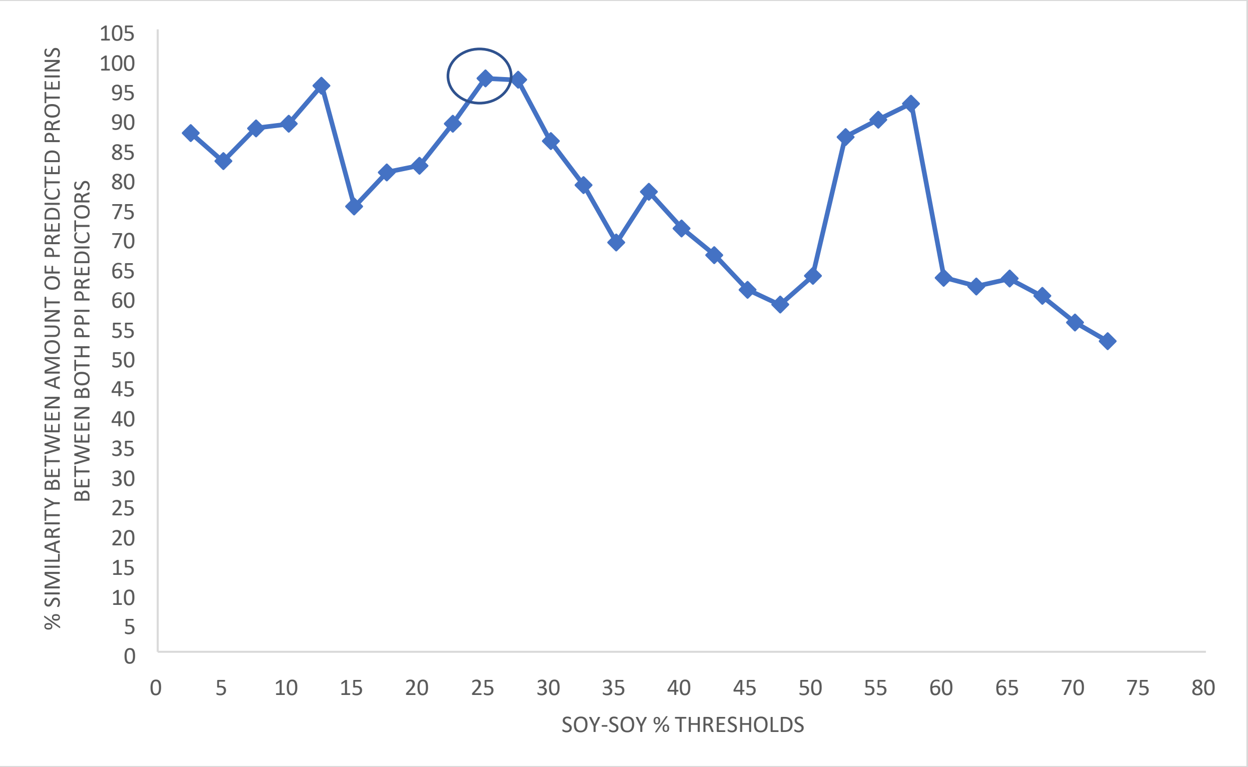

Supplementary Figure 1. Scatter plot of percent similarity between both PPI predictors and the soy-soy percent thresholds. Highlighted in the gray circle is the highest percent similarity of predicted top interactors between PIPE4 and SPRINT shown to be at 25% soy-soy threshold.

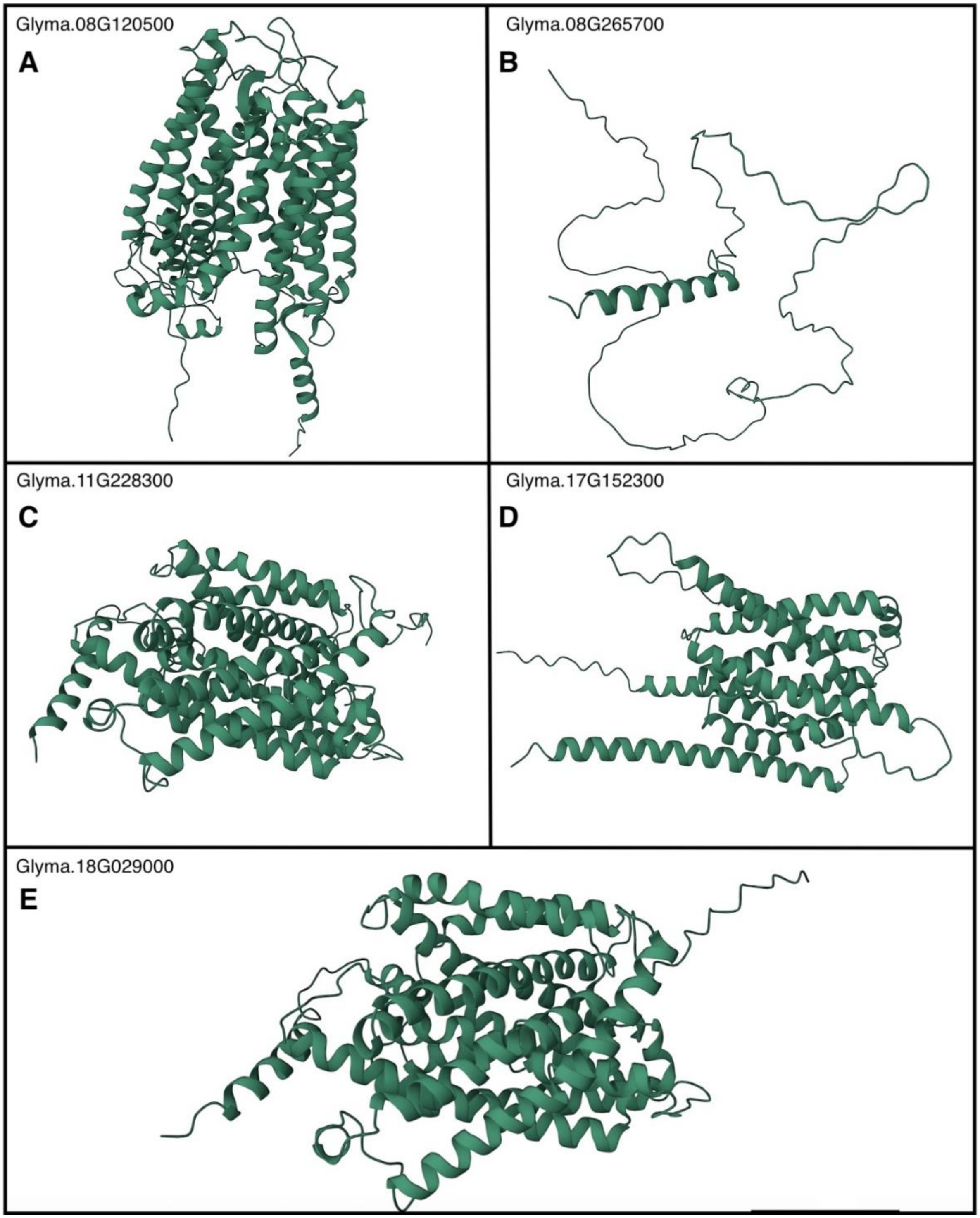

Supplementary Figure 2. AlphaFold2 generated depictions of the five most relevant predicted proteins which were involved in response to nematode GO.
